# Histone H2B induces retinal ganglion cell death through toll-like receptor 4 in the vitreous of acute primary angle closure patients

**DOI:** 10.1038/s41374-020-0427-2

**Published:** 2020-04-22

**Authors:** Yasunari Munemasa

**Affiliations:** Department of Ophthalmology, St. Marianna University School of Medicine, 2-16-1 Sugao Miyamae, Kawasaki, Kanagawa 2168511 Japan

**Keywords:** Proteolysis, Translational research

## Abstract

Acute primary angle closure (APAC) is a disease of ophthalmic urgency; lack of treatment can lead to blindness. Even after adequate treatment for APAC, subsequent elevated acute intraocular pressure induces severe neuronal damage which can result in secondary glaucomatous optic neuropathy (GON). Damage-associated molecular patterns (DAMPs) are released from damaged and dead neuronal cells, which induce secondary inflammatory changes and further tissue damage. Our hypothesis is that histone H2B (H2B), which is one of the DAMPs, is released from damaged cells in the development of GON after APAC treatment. Intravitreal injection of H2B induces neuronal cell death through toll-like receptor 4 (TLR4) expression, following the upregulation of inflammatory cytokine mRNAs and phosphorylation of mitogen activated protein kinases (MAPKs). Knockdown of TLR4 caused a reduction of H2B neurotoxicity in damaged cells through TLR4 signaling. Significantly increased H2B was observed in the vitreous cells of APAC patients. In addition, enhanced H2B protein correlated with decreased ganglion cell analysis and retinal ganglion cell (RGC) layer thinning, which indicates the effect of H2B on RGCs. Our data from clinical and animal studies show the involvement of H2B-TLR4 pathways in the development of GON after APAC treatment providing new insight for the mechanism of RGC degeneration.

## Abstract (web summary)

Increased levels of histone H2B (H2B), a damage-associated protein, are found in the vitreous of patients with acute primary angle closure. Elevated H2B causes severe inflammation and subsequent retinal ganglion cell death through toll-like receptor 4 (TLR4) signaling. These results provide new insight for the mechanism of retinal ganglia degeneration.

## Introduction

Toll-like receptors (TLRs) are type I transmembrane proteins containing extracellular leucine-rich repeats (LRR) and cytoplasmic toll/intracellular interleukin (IL)-1 receptor (TIR) domains similar to that of the IL-1 receptor [1, 2]. TLRs are stimulated by binding various ligands, such as lipoproteins, peptidoglycans, microbial molecules, and lipopolysaccharides to LRR [3–5]. Homo- or heterodimers of these components stimulate the production of several adapter molecules, including myeloid differentiation primary response gene 88 (MyD88), TIR-domain-containing adapter-inducing interferon-β (TRIF), and the TRIF-related adapter molecule (TRAM). Subsequent to the production of these molecules, several inflammatory reactions and immune responses are triggered [6, 7]. Activation of TLRs is reported in retinal ganglion cell (RGC) degeneration in response to axotomy of the optic nerve, ischemia-reperfusion injury, and excessive glutamate production in the retina [8]. Nuclear factor kappa-light-chain-enhancer of activated B cells p65, mitogen activated protein kinases (MAPKs), and interferon regulatory factor 3, which are downstream of MyD88, TRIF, and TRAM, each contribute to RGC degeneration [9–11].

The pattern of RGC degeneration in glaucomatous optic neuropathy (GON) is varied. Chronic RGC degeneration is observed in most patient cases of open angle glaucoma (OAG) and normal tension glaucoma (NTG) [12]. Previous study indicates that the development of GON is due to the deformation of lamina and the remodeling of the connective tissues in the surrounding peripapillary sclera [13]. In contrast, acute primary angle closure (APAC) induces relatively acute RGC death, showing that the optical coherence tomography (OCT) finding confirms that RGC axonal damage in APAC patients is due to nerve fiber layer thinning at a relatively early period after APAC treatment [14]. Although several studies have established the mechanism of chronic RGC death in OAG and NTG, the mechanism for acute RGC death in APAC has not been completely resolved. Intraocular pressure (IOP) elevation in APAC causes increased cytokine production in the aqueous humor, including granulocyte colony-stimulating factor, IL-6, IL-8, monocyte chemotactic protein (MCP)-1, and vascular endothelial growth factor [15]. Though these changes take place in the anterior chamber, it does not completely explain the development of axonal degeneration in APAC. Therefore, it is critically important to analyze molecular changes occurring in the vitreous humor and retinas in APAC patients.

Damage-associated molecular patterns (DAMPs) released from dead and damaged cells enhance the inflammatory response and subsequent tissue injury. Although several DAMPs in the human vitreous are reported in retinal neuronal diseases such as retinal detachment, proliferative vitreoretinopathy, and diabetic retinopathy, the DAMPs related to the development of GON have not been elucidated [16]. Histones are highly alkaline nuclear proteins that compact DNA strands and are involved in chromatin regulation. Histones are released from apoptotic and necrotic cells or actively secreted by activated inflammatory cells in the form of neutrophil extracellular traps (NETs) [17, 18]. It is commonly known that histone is one of ligands of toll-like receptor 4 (TLR4) [19]. Cytokines released during histone docking of TLR4 cause inflammatory reactions and subsequently induce cell death through MyD88, TRIF, and TRAM pathways [20].

In this study, we hypothesized that DAMPs released from dying cells in response to acute elevated IOP with APAC induces secondary cell death via TLR4 pathways. Here, we attempt to qualitatively detect DAMPs in the vitreous in patients with APAC and to investigate the role of DAMPs both in vivo and in vitro.

## Materials and methods

### Animals and surgical procedures

The use of animals was approved by the Animal Research Committee of St. Marianna University and the study was performed in compliance with the ARVO Statement for the Use of Animals in Ophthalmic and Vision Research. Male C57BL/6J TLR4^−/−^ mice were purchased from Oriental Bio Service Adult (Kyoto, Japan). The animal room was lit with fluorescent lights (330 lux) with a 12-h light/dark cycle, and the temperature was kept at 21 °C. The animals were allowed to acclimate to this environment for at least 1 week before any surgical procedures. Mice were anesthetized with an intramuscular injection of a mixture of ketamine–xylazine (10 and 5 mg/kg, respectively). Intravitreal injections of 30 or 300 μmol H2B were performed under a limbus microscope set at 1 μm. Retrograde labeling of RGCs was performed, as described previously [21]. Labeling was performed at 7 days before the intravitreal injection procedure.

### Immunoprecipitation

One day after the intravitreal injection of H2B, membrane proteins were extracted from retinal cell lysis with a subcellular proteome extraction kit (Calbiochem, Darmastadt, Germany) according to the manufacturer’s instructions. Immunoprecipitation was carried out with the Immunoprecipitation Starter Pack (GE Healthcare Bio-Sciences AB, Uppsala, Sweden), as described previously [22]. The supernatants were incubated with a TLR4 antibody (Santa Cruz Biotechnology, Santa Cruz, CA). Proteins were separated on a 4–20% SDS-PAGE gel (Bio-Rad, Hercules, CA), and then subjected to silver staining (Thermo Scientific, Waltham, MA).

### Pull-down (tag based) assay

The immunoprecipitation and pull-down assay were employed to detect the precipitated protein complexes and investigate the interaction between the proteins. Expression of N-terminal His-tagged histone H2B (His-H2B, BPS Bioscience, San Diego, CA) was confirmed by SDS-PAGE and silver staining. Overexpression of TLR4 was generated in PC12 cells using plasmid DNA (sense 5′-AGTGGGTCAAGGAACAGAAGCA-3′ (forward) and 5′-CTTTACCAGCTCATTTCTCACC-3′ (reverse)) and lipofectamine 2000 to increase the DNA transfection rates. TLR4 overexpressed cells were incubated overnight with equal amounts of His-H2B proteins at 4 °C. The proteins that bound to the magnetic beads were eluted with SDS sample buffer and analyzed by SDS-PAGE and immunoblotting. Immunoblotting was performed with the His-tag antibody (Cell Signaling, Danvers, MA).

### Immunohistochemistry

Mice that received the intravitreal injection were *trans*-cardially perfused with 4% paraformaldehyde in 0.1 M phosphate buffer. The eyes were enucleated 1 day after intravitreal injection of either H2B or PBS. 5-μm–thick sections were incubated with primary antibodies against TLR4 and Iba-1 (marker for microglia, WAKO, Osaka, Japan) and were incubated with the secondary rhodamine-conjugated anti-rabbit IgG antibody (Cappel Research Products, Durham, NC).

### Immunoblotting

Immunoblotting was performed as previously described [23]. Retinas from mice were collected 1 day after the intravitreal injection. Membranes were first reacted with anti-β-actin (Sigma, St. Luis, MO), anti-p-ERK, ERK, p-JNK, JNK, p-P38, and P38 antibody (1:200; cell signaling).

### Real-time polymerase chain reaction (PCR)

The quantitative reverse-transcription PCR Applied Biosystems (Life Technologies, Tokyo, Japan) protocol was used. One day after the intravitreal injection, retinas were collected, and total RNA was isolated using ReverTra Ace (TOYOBO, Osaka, Japan). The expression of β-actin was used for normalization of variation between the levels of total cDNA template across different samples. The primers for IL-1β were 5′-CGAGGCTAATAGGCTCATCT-3′ (forward) and 5′-GTTTGGAAGCAGCCCTTCAT-3′ (reverse), with a 177 bp amplicon (GenBank Accession No. XM_006498795). The primers for tumor necrosis factor alpha (TNF-α) were 5′-CTACTCCCAGGTTCTCTTCAA-3′ (forward) and 5′-GCAGAGAGGAGGTTGACTTTC-3′ (reverse), with a 118-bp amplicon (GenBank Accession No. NM 001278601.1). The primers for transforming growth factor beta (TGF-β) were 5′-CTCCCGTGGCTTCTAGTGC-3′ (forward) and 5′-GCCTTAGTTTGGACAGGATCTG-3′ (reverse), with a 133-bp amplicon (GenBank Accession No. NM_ 011577.2). Serial dilutions of the standard templates were also used for parallel amplifications. The primers for MCP1 were 5′-CCCCACTCAGCTGCTACT-3′ (forward) and 5′-GCCATCACACTCGTCACA-3′ (reverse). The primers for β-actin were 5′-AACACCCCAGCCATGTAC-3′ (forward) and 5′-ATGTCACGCACGATTTCCC-3′ (reverse), with a 133-bp amplicon (GenBank Accession No. NM_ 007393.5). The threshold cycles were calculated with Step One Plus Real-time PCR system (TOYOBO). Standard curves were plotted with threshold cycle-versus-log template quantities.

### Morphology

#### Morphometry of cells in the ganglion cell layer (GCL)

The morphometry of neural cells in the GCL on whole-mounted and flat preparations of retinas was performed as described previously [21]. Eyes were obtained from mice at 7 days after the intravitreal injection. The retinas were stained with 1% cresyl violet. RGC or neuronal cells counting was performed in eight distinct areas, that is, 500 and 1000 μm from the edge of the optic disc in each retinal quadrant (nasal, temporal, inferior, and superior). Data from eight areas of each eye were averaged for one eye. Quantification was performed in a blinded manner [21].

### Retrograde labeling of retinal ganglion cells

Retrograde labeling of RGCs was performed, as described previously [21]. Eyes were obtained 7 days after intravitreal injection and processed in flat-mount preparations. Dye-labeled RGCs were counted at 0.4 and 0.8 mm from the center of the optic nerve in the retinal quadrant under fluorescence microscopy (Axioskop; Carl Zeiss, Oberkochen, Germany) at 200× magnification. Quantification was performed in a masked manner.

### Cell culture and transfection

PC12 cells were graciously provided by Prof. Ohtani-Kaneko of Toyo University Life and Science. The cells were maintained in high glucose Dulbecco’s modified Eagle’s medium (Invitrogen, Carlsbad, CA) supplemented with 10% fetal bovine serum (FBS, Invitrogen), 100 units/mL penicillin, and 100 mg/mL streptomycin. Cells were maintained in humidified 5% CO_2_ and 95% air environment at 37 °C, as described previously. Twenty-four hours before transfection, exponentially growing cells were harvested by trypsinization and replated at a density of 1 × 10^5^ cells/cm^2^ with appropriate medium. The lipofectamine 2000 (Invitrogen) mediated transfection procedure was used to introduce pEGFP-C1-TLR4 plasmid DNAs into the PC12 cells. These constructs were prepared by cloning of human TLR4 cDNA into pEGFP-C1. Transcription of the TLR4 genes in each vector is controlled by the cytomegalovirus promoter. The cells were also treated with either 50 nM TRIF siRNA (Ambion) or 50 nM Myd88 siRNA (Ambion) with lipofectamine (Invitrogen) without any other treatments.

Cell viability was determined by cell proliferation colorimetric assay kit (WST assay, BioVision, Milpitas, CA), as stated in the manufacturer’s protocol. Cells were seeded on a 96-well plate and were treated with 5 or 10 mM glutamate (Fisher Scientific, Chino, CA) and 0.5 mM BSO (Sigma), which inhibits glutamate cysteine ligase.

### Enzyme-linked immunosorbent assay (ELISA)

ELISA for H2B levels was performed as per manufacturer instructions (Cloud-Clone Corporation, Houston, TX).

### Inclusion and exclusion criteria of APAC

APAC is defined as follows: (a) IOP elevation of at least 30 mmHg and typical signs such as ocular pain, nausea, and vomiting, (b) clinical signs involved in conjunctival injection, microcytic corneal edema, mid-dilated pupil, and shallow AC, (c) presence of angle closure, confirmed by gonioscopy. Angle closure was defined referring AAO guidelines (https://www.aao.org/preferred-practice-pattern/primary-angle-closure-ppp-2015) as iridotrabecular contact more than 180 degree.

Eyes with iris or angle neovascularization, pseudoexfoliation, lens subluxation, any iris or corneal abnormalities were excluded.

### Analysis of human vitreous samples

We performed a retrospective review of patients with APAC or with an idiopathic epiretinal membrane (iERM) condition who underwent surgery at St. Marianna University Hospital, between April 1, 2012 and March 31, 2015. This study was approved by the Ethics Committee of St. Marianna University Hospital and was performed in accordance with the Helsinki Declaration. All patients provided informed consent before surgery. Diagnosis of APAC was by slit lamp, Goldmann tonometer, and anterior segment OCT (CASIA Tomey, Nagoya Japan). Combined anterior 25-gauge (G) pars planner vitrectomy (PPV) and phacoemulsification and aspiration with intraocular lens were performed on all patients. A volume of 0.5–0.7 mL vitreous sample was collected from the anterior vitreous by PPV and centrifuged to remove cellular debris and cells. Vitreous samples collected by 25 G PPV for ERM were used as control samples. The pellets from the centrifugation were discarded and the supernatant from each sample was stored at −80° until use. Measurement of vitreous levels of H2B was determined by ELISA. The correlation of concentration of vitreous H2B and integral IOP (pressure × duration), which is represented by the duration of elevated IOP before surgery from onset of APAC, was statistically analyzed.

### Optical coherence tomography analysis

Cirrus HD-OCT (Carl Zeiss Meditec, Dublin, CA) was used to acquire one macular (Macular Cube 200 × 200 protocol) and one optic disc (Optic Disc Cube 200 × 200 protocol) scan in each qualifying eye, after pupil dilation with 1% tropicamide and 2.5% phenylephrine. The ganglion cell analysis (GCA) algorithm used to process these data from 6.0 software version of Cirrus OCT; the algorithm detects and measures the thickness of the macular GCIPL within a 14.13-mm^2^ elliptical annulus area centered on the fovea. The size and shape of the annulus was chosen because it conforms more closely to actual anatomy, and this annulus corresponds to the area where the RGC layer (RGCL) is thickest in normal eyes. The GCA algorithm processes data from three-dimensional volume scans using the Cirrus macular 512 × 128 × 1024 or 200 × 200 × 1024 acquisition protocol. Processing is performed in three dimensions. Data were obtained 1 month and 12 months after surgery. Changes in thinning of retinal neurofiber layer (RNFL) and GCA at 12 months post surgery were compared with those noted at 1 month.

### Statistical analysis

Data are presented as the mean ± SD. Correlations of H2B and integral IOP or RGC damage, such as GCA and RNFL thinning shown by OCT, were analyzed by simple linear regression analysis and Spearman’s rank correlation coefficient. Differences among groups were analyzed by one-way ANOVA, followed by the Scheffe’s or Mann–Whitney *U* tests. A *p* value of < 0.05 was considered statistically significant.

## Results

### Expression of TLR4 in the retina after intravitreal injection of H2B

**Fig. 1:**
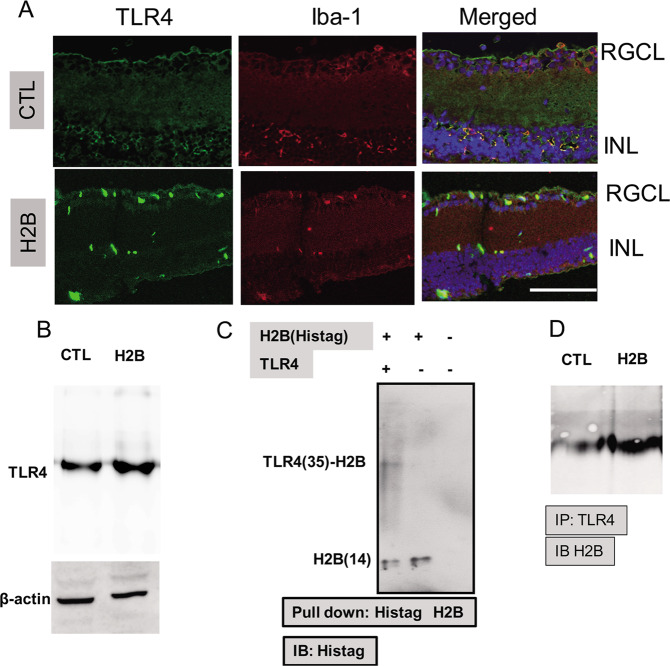
Immunohistochemistry, Immunoblot, and Pull-down assay. Immunohistochemistry for TLR4 in wild-type mice retina after the intravitreal injection of H2B (**a**). Iba-1 was used for a maker of microglia cells. RGCL retinal ganglion cell layer, INL inner nuclear layer. Immunoblot for TLR4 in the wild-type retina after the intravitreal injection of H2B (**b**). Pull-down assay using His-tag Histone H2B and TLR4 overexpressed PC12 cells (**c**). Immunoblot was performed with His-tag antibody (**c**). Immunoprecipitation with TLR4 antibody and immunoblot with H2B antibody (**d**).

Double immunofluorescence showed that the colocalization of TLR4 was observed in Iba-1 positive cells, a marker for microglia, in the inner retina at 7 days after intravitreal injection of H2B. This result implies that the effect of H2B predominantly occurred in the inner retina (Fig. 1a). These results were confirmed by immunoblot, showing that increased TLR4 was observed in the retina after intravitreal injection of H2B (Fig. 1b).

### Interaction of TLR4 with H2B

To confirm that H2B is one of the ligands of TLR4, a pull-down assay with a His-tag antibody was performed. Two bands with molecular weights of ~50 and 15 kDa were detected by immunoblotting with the His-tag antibody following tagged based pull-down assay (Fig. 1c). These results were consistent with the in vivo study that observed elevated H2B levels in the retina treated with intravitreal injection of H2B after IP with TLR4 antibody (Fig. 1d).

### Neurotoxicity of H2B through TLR4 in the retina

**Fig. 2:**
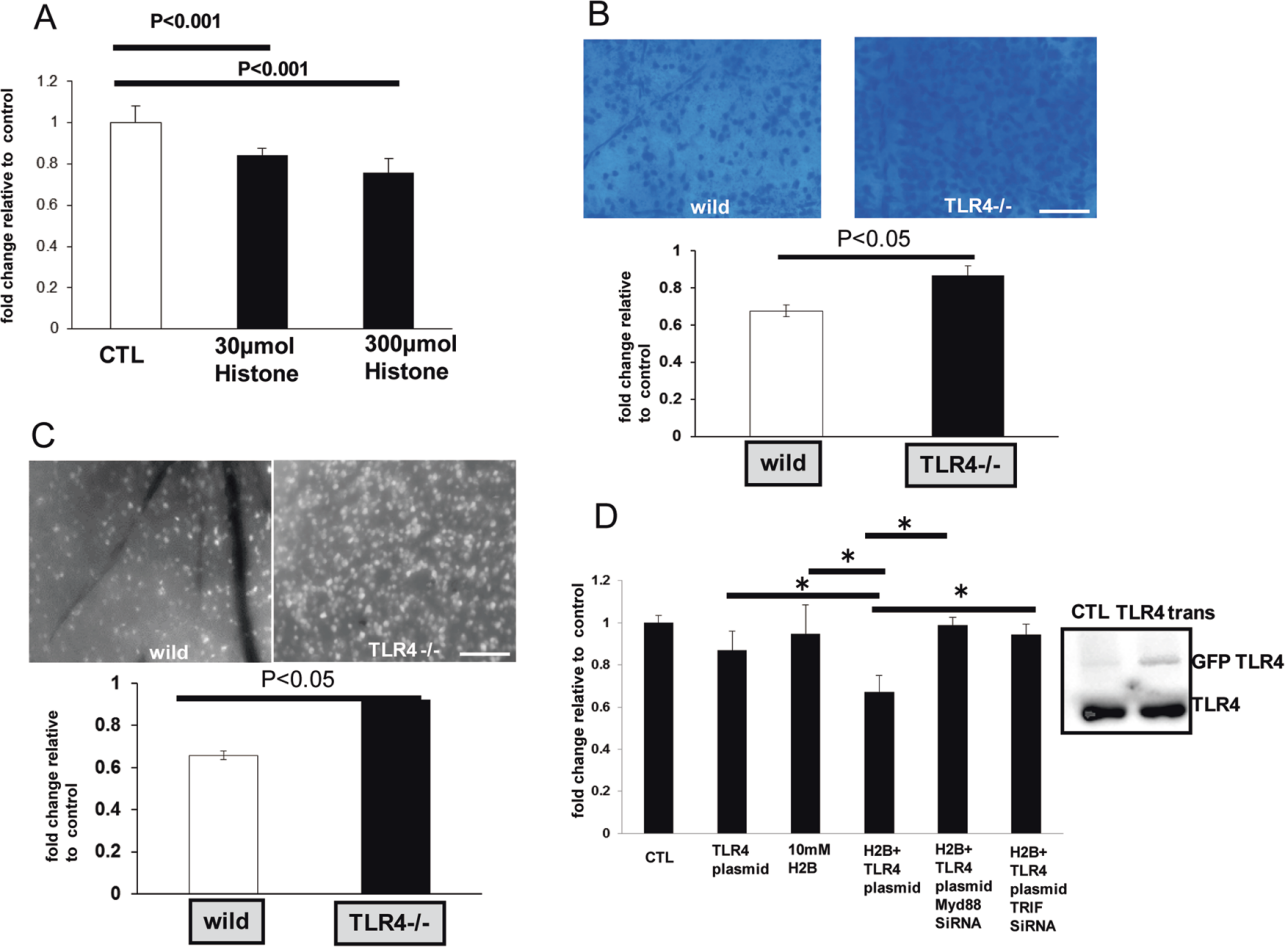
Morphology. Morphological analysis of neuronal cells (amacrine cells and RGCs) in the RGCL of wild-type mice after the intravitreal injection of H2B (30 or 300 µmol) by cresyl violet staining (**a**). Photomicrograph for wholemount retina of wild or TLR4 knockout mice after the intravitreal injection of H2B stained with cresyl violet (**b**). Scale bar is 100 µm. Fluorescence photomicrograph for FG labeled RGCs of wild or TLR4 knockout mice after intravitreal injection of H2B (**c**). Viability of PC12 cells for TLR4 overexpression with plasmid DNA and H2B or siRNA of TRIF or Myd88 (**d**).

Two types of neuronal cells in the RGCL, RGCs, and amacrine cells were stained with cresyl violet, which can distinguish between the two types of cells and confirm the effect of H2B. Fluorogold labeling was retrogradely performed to identify RGCs. The intravitreal injection of H2B (30 or 300 µmol) induced neurotoxicity in the RGCL of wild-type (C57BL/6J) mice 7 days after the injection, with ~30% neuronal cell loss, compared with the phosphate buffer saline control (*n* = 5, *p* < 0.05) (Fig. 2a). In contrast, a change in H2B burden was not observed in the RGCL of TLR4^−/−^ mice (*n* = 5, *p* < 0.05) (Fig. 2b). Consistent with the results of cresyl violet staining, a neurotoxic effect of H2B was not observed in TLR4^−/−^ mice, but RGC injury by H2B intravitreal injection a loss of ~35% loss in neuronal cells from wild-type mice (*n* = 5, *p* < 0.05) (Fig. 2c). The neurotoxic effect of H2B was also studied in vitro using PC12 cells. Although the treatment of H2B (10 mM) did not show a toxic effect, overexpression of TLR4 with plasmid DNA exerted an H2B effect on PC12 cells (*n* = 6, *p* < 0.05) (Fig. 2d). Treatment with Myd88 or TRIF siRNA, which are effector molecules downstream of TLR4, impaired the effect of H2B through TLR4 overexpression (Fig. 2d).

#### Change in pro-inflammatory cytokines after intravitreal injection of H2B

**Fig. 3:**
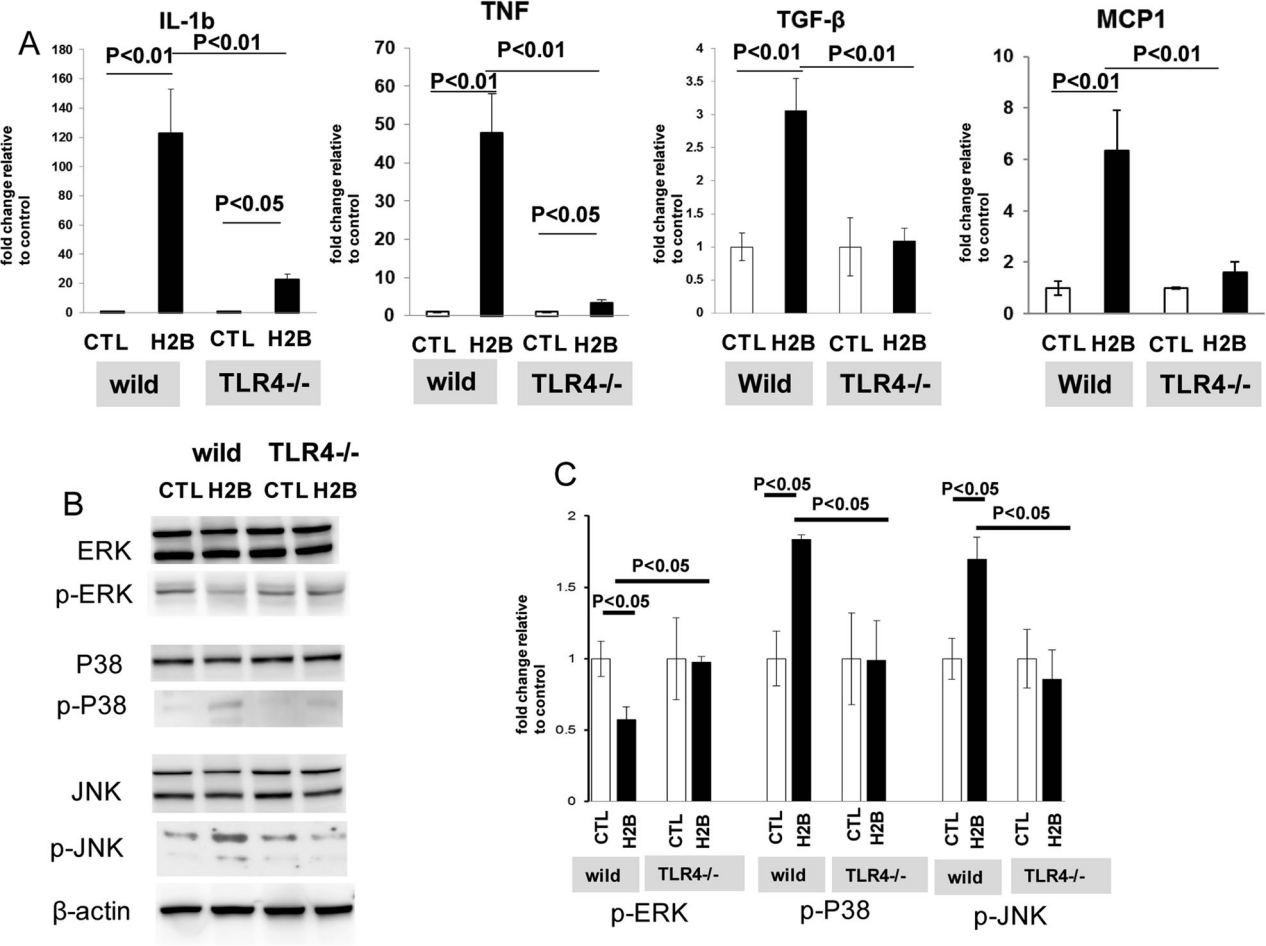
Real time PCR and semi-quantitative Immunoblot. Real-time PCR for inflammatory cytokines (**a**). Immunoblot for phosphorylation of MAPKs (ERK, p38, and JNK) (**b**). Densitometry for immunoreactive bands for MAPKs. Data are normalized to β-actin levels in each sample (**c**).

Real-time PCR was used to study the change of mRNA of pro-inflammatory cytokines, such as IL-1β, TNF, TGF-β, and MCP1, in H2B-treated retinas. Dramatic upregulation of IL-1β (*n* = 5, *p* < 0.01), TNF-α (*n* = 5, *p* < 0.01), TGF-β (*n* = 5, *p* < 0.01), and MCP1 (*n* = 5, *p* < 0.01) mRNA was observed in wild-type retinas after intravitreal injection of H2B (Fig. 3a). Because significant upregulation of IL-1β (*n* = 5, *p* < 0.05) and TNF-α (*n* = 5, *p* < 0.05) was observed in the TLR4^−/−^ retinas after intravitreal injection of H2B, these changes were significantly attenuated in the TLR4^−/−^ retinas (*n* = 5, *p* < 0.01) (Fig. 3a). Phosphorylation of MAPKs is related to retinal neuronal apoptotic cell death [24]. Therefore, we performed immunoblotting to study the change in phosphorylation of MAPKs after intravitreal injection of H2B. Significant phosphorylation of JNK and p38 was observed in the wild-type mice retinas after the intravitreal injection of H2B, as compared with the controls (*n* = 4, *p* < 0.05) (Fig. 3b, c). On the contrary, dephosphorylation of ERK was observed in the H2B-treated retinas (*n* = 4, *p* < 0.05). These molecular changes were attenuated in the retinas of TLR4^−/−^ after the intravitreal injection of H2B, showing that TLR4 knockdown contributes to the inhibition of antiapoptotic cell death (Fig. 3b, c).

#### H2B levels in the vitreous of patients with APAC

**Fig. 4:**
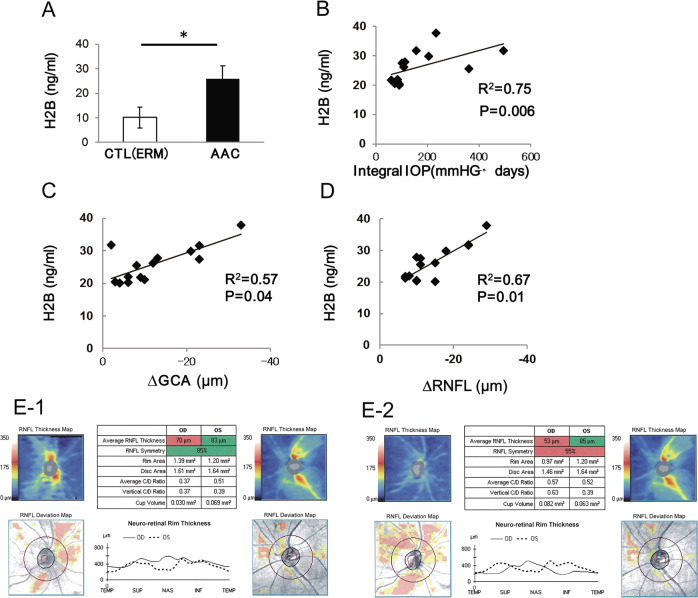
ELIZA and OCT. Concentration of H2B in patients with APAC and control (iERM) (**a**). Correlation of H2B concentration and integral IOP (pressure × duration) (**b**). Correlation of H2B concentration and ΔGCA (change of GCA at 1 and 12 M after the surgery) (**c**). Correlation of H2B concentration and ΔRNFL (change of RNFL at 1 and 12 M after the surgery) (**d**). Representative OCT for one case of APAC patient at 1 M (E1) and 12 M (E2) after the surgery.

Demographic characteristics of the APAC and control groups are presented in Table 1. The specific diagnoses of APAC was noted in 14 patients and 23 iERM patients served as a control group. Significantly elevated H2B was detected in the vitreous of APAC patients (*p* < 0.05). No significant differences in average age (*p* > 0.05) and sex (*p* > 0.05) were observed in iERM and APAC (Table 1). A significant increase of H2B was observed in the vitreous of patients with APAC, as compared with iERM patients, with an approximately 2.5-fold change in APAC patients (iERM patients’ concentration of 10.14 ng/mL as compared with 25.98 ng/mL in APAC patients). The concentrations of H2B in APAC patients were correlated with integral IOP, which is expressed as IOP multiplied by duration (days) of elevated IOP (*n* = 14, *R*^2^ = 0.75, *p* = 0.006) (Fig. 4b). Analysis of OCT showed that decreased RNFL and GCA were observed in APAC patients at 1 year after surgery (Fig. 4c, d). The concentration of H2B was correlated with decreased GCA (*n* = 14, *R*^2^ = 0.57, *p* = 0.04) and RNFL thickness (*n* = 14, *R*^2^ = 0.67, *p* = 0.01) (Fig. 4c, d), indicating the impact of H2B on the RGC. The change for OCT in a representative patient case is shown in Fig. 4e-1 and e-2. Figure 4e-1 is 1 month after surgery with an RNFL of 70 µm, with thinning of RNFL thickness to ~17 µm at 12 months after surgery (Fig. 4e-2).

**Table 1:** Demographic for control (iERM) and APAC group.

	CTL (ERM)	APAC	*P*
Number of patients	23	14	
Number of eyes	23	14	
Age (years)	72.8 ± 8.1	74.3 ± 1.8	0.754^a^
Gender, *n* (%)			
Male	39.1%	21.4%	0.059^b^
Female	60.9%	78.6	
Disease duration (days)	None	3.2 ± 0.6	
Integral IOP (IOP × disease duration, mmHg days)	None	160.5 ± 35.9	

^a^Mann–Whiteny *U* test.

^b^*χ*^2^ test.

**Fig. 5:**
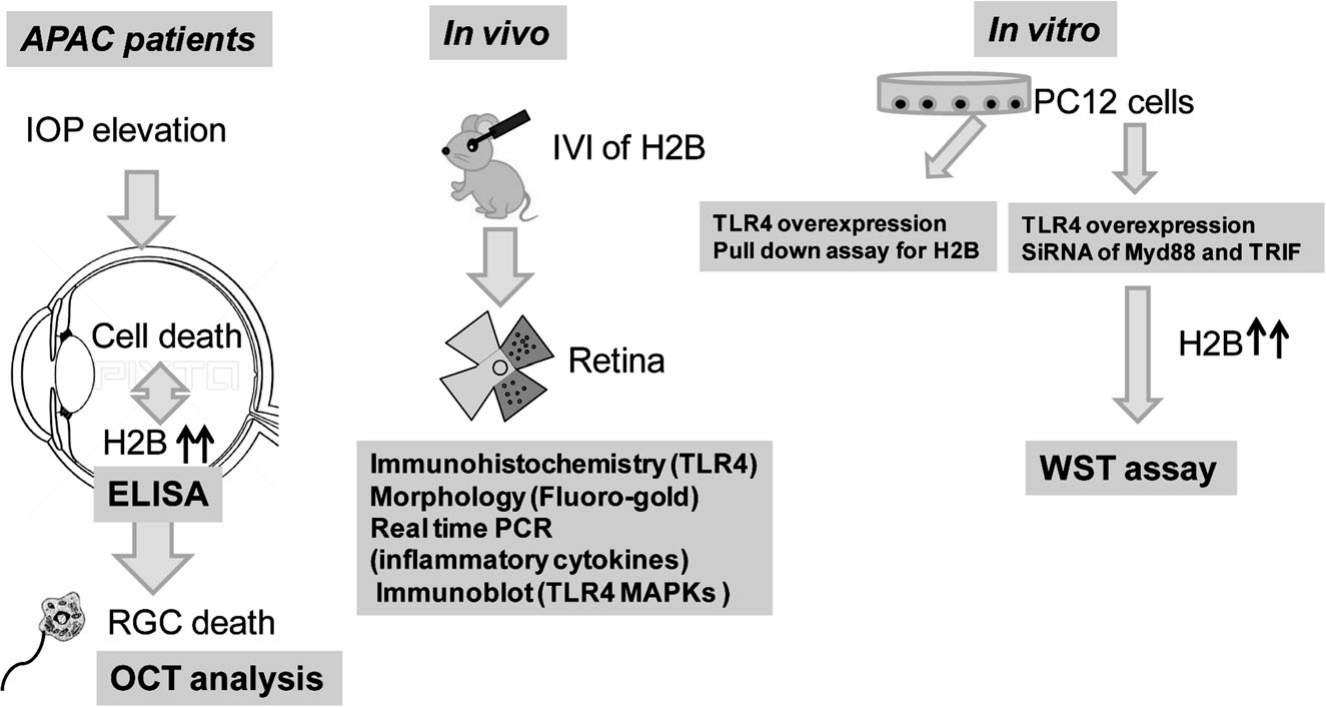
Flow chart of this study.

Overall flow of this study is shown in schema as Fig. 5.

## Discussion

Histones can be passively released from necrotic or apoptotic cells and can activate inflammatory cells in the formation of NETs [18]. Elevated NETs formation induces thrombophilia and degradation of cell organelles during various clinical conditions, such as sepsis, aggravated kidney injury, trauma, and autoimmune disease [25]. Extracellular histones with NETs induce cytotoxic damage in endothelial cells, platelets, and neurons and were recognized as DAMPs [26]. Elevated histone levels are found in seriously ill patients with cerebrovascular diseases, ischemic heart diseases, autoimmune diseases, and traumas and other conditions [27–30]. In the present study, we report elevated H2B, which is correlated with integral IOP, in the vitreous of patients with APAC, indicating the impact of extracellular H2B concentration on IOP elevation. OCT analysis showed that elevated H2B induces RGC injury. The release of H2B due to IOP elevation produces neurotoxicity in RGC in APAC patients.

Previous studies report distinct receptor-dependent and receptor-independent mechanisms of extracellular histones, which can function separately, or in concert to induce tissue injury [31]. In the receptor-dependent mechanism, histones bind to and activate TLRs on various cells, which subsequently triggers inflammation or platelet aggregation [31–33]. Alternatively, histone-mediated cytotoxicity through receptor-independent means is due to a positive charge, which enables direct binding to the negatively charged plasma membrane of target cells [34, 35]. In the present study, although PC12 cells with treatment of H2B alone did not show neurotoxicity, PC12 cells with TLR4 overexpression showed vulnerabilities against H2B treatment. In addition, knockout of downstream molecules of the TLR4 pathway by siRNA (TRIF and Myd88) inhibited the influence of H2B, indicating that H2B neurotoxicity is mediated in a TLR4 receptor-dependent manner.

Our immunoblot and immunohistochemistry showed activation of TLR4 in the retina after the intravitreal injection of H2B, especially in Iba-1 positive microglia. These results are consistent with previous studies that show the activation of TLR4 in retinal injuries in immune cells, such as microglia [35]. Intravitreal injection of H2B induced amacrine and RGC death in the RGCL. Our qPCR indicated dramatic upregulation of pro-inflammatory cytokines, especially IL-1β and TNF-α, after the intravitreal injection of H2B. In addition, phosphorylation of p38 and JNK was observed in the retinas treated with H2B. In contrast, dephosphorylation of ERK was noted in H2B-treated retinas. I propose that these changes may contribute to RGC injury. The mechanisms of histone cytotoxicity are reported as various types of cytotoxicity, including induction of vascular permeability, coagulation activation, platelet aggregation, and cytokine production [36, 37]. Our in vivo study also showed the induction of pro-inflammatory cytokines and subsequent phosphorylation of pro-apoptotic molecules, such as p38 and JNK, by H2B treatment. TLR4 knockdown exerted these changes, indicating that the effect of H2B is mediated through TLR4 pathways.

IOP is occasionally elevated more than ocular blood flow pressure during high IOP with APAC. I therefore proposed that H2B is released from various hypoxic and damaged cells, such as iris pigment epithelium cells, ciliary epithelium cells, vascular endothelial cells, and retinal glial or neuronal cells. During hypoxic conditions, TLR4 is activated and contributes to neuronal death through inflammation [38]. Therefore, in the present study, the H2B released from various hypoxic cells induced additional inflammation and subsequently led to retinal neuronal cell death through TLR4 pathways.

In summary, the present study provides new insight for the mechanism of RGC degeneration in APAC with the involvement of H2B-TLR4 pathways. Modulation of TLR4 during elevated IOP in APAC patients may be useful as a potential therapy for the prevention of predicted RGC damage.
